# MicroRNA and mRNA Expression Changes in Glioblastoma Cells Cultivated under Conditions of Neurosphere Formation

**DOI:** 10.3390/cimb44110360

**Published:** 2022-10-30

**Authors:** Maya A. Dymova, Natalia S. Vasileva, Elena V. Kuligina, Yulya I. Savinovskaya, Nikita D. Zinchenko, Alisa B. Ageenko, Sergey V. Mishinov, Grigory A. Stepanov, Vladimir A. Richter, Dmitry V. Semenov

**Affiliations:** 1 Institute of Chemical Biology and Fundamental Medicine, Siberian Branch, Russian Academy of Sciences, Lavrentyev Avenue, 8, 630090 Novosibirsk, Russia; 2 Novosibirsk Research Institute of Traumatology and Orthopedics n.a. Ya.L. Tsivyan, Department of Neurosurgery, Frunze Street 17, 630091 Novosibirsk, Russia

**Keywords:** glioblastoma, cancer stem cells, neurospheres, epithelial to mesenchymal transition, pro-neural to mesenchymal transition, RNA-seq, miRNA, mRNA

## Abstract

Glioblastoma multiforme (GBM) is one of the most highly metastatic cancers. The study of the pathogenesis of GBM, as well as the development of targeted oncolytic drugs, require the use of actual cell models, in particular, the use of 3D cultures or neurospheres (NS). During the formation of NS, the adaptive molecular landscape of the transcriptome, which includes various regulatory RNAs, changes. The aim of this study was to reveal changes in the expression of microRNAs (miRNAs) and their target mRNAs in GBM cells under conditions of NS formation. Neurospheres were obtained from both immortalized U87 MG and patient-derived BR3 GBM cell cultures. Next generation sequencing analysis of small and long RNAs of adherent and NS cultures of GBM cells was carried out. It was found that the formation of NS proceeds with an increase in the level of seven and a decrease in the level of 11 miRNAs common to U87 MG and BR3, as well as an increase in the level of 38 and a decrease in the level of 12 mRNA/lncRNA. Upregulation of miRNAs hsa-miR: -139-5p; -148a-3p; -192-5p; -218-5p; -34a-5p; and -381-3p are accompanied by decreased levels of their target mRNAs: RTN4, FLNA, SH3BP4, DNPEP, ETS2, MICALL1, and GREM1. Downregulation of hsa-miR: -130b-5p, -25-5p, -335-3p and -339-5p occurs with increased levels of mRNA-targets BDKRB2, SPRY4, ERRFI1 and TGM2. The involvement of SPRY4, ERRFI1, and MICALL1 mRNAs in the regulation of EGFR/FGFR signaling highlights the role of hsa-miR: -130b-5p, -25-5p, -335-3p, and -34a-5p not only in the formation of NS, but also in the regulation of malignant growth and invasion of GBM. Our data provide the basis for the development of new approaches to the diagnosis and treatment of GBM.

## 1. Introduction

In the general structure of oncological diseases, glioma is a rather rare disease; however, it is one of the most common primary brain tumors, and leads to significant morbidity and mortality. It is characterized by a variable course of the disease and prognosis depending on the type [1]. Of all six types and grades of glioma, glioblastoma (GBM) is the most aggressive, an incurable and common (48.60%), with a 5-year survival rate of less than 5% and a median survival of approximately 15 months under full standard treatment [2,3]. The reasons for such a low survival rate should be sought in both tumor heterogeneity, propensity with brain invasion, immune evasion, presence of natural barriers, as well as cancer stem cells (CSCs) [4,5,6,7]. The latter represent a big hurdle for glioblastoma treatment due to their involvement in drug and radiation resistance, unlimited potential for self-renewal and for multilineage differentiation as well. Thus, the development of new approaches to the diagnosis and therapy of glioblastoma remains an urgent task, for which the most relevant cell models are needed.

The neurosphere assay quickly became the method of choice and has since become a valuable tool for isolating and understanding the biology of both embryonic and adult central nervous system (CNS) stem cells and cancer stem cells [8], unconditionally, with all the limitations of the method [9]. The formation of neurospheres from monolayer (MN) cultures of glioma cells is carried out under conditions of cell cultivation in a serum-free medium in the presence of epidermal growth factor (EGF) and basic fibroblast growth factor (bFGF). During the formation of neurospheres, adherent cell cultures undergo a number of changes in the transcriptome, metabolome, proteome, which must be taken into account when interpreting the data of the corresponding study [10,11,12]. However, regulatory RNAs, such as miRNAs, have received little attention in such studies. Although recently, interest in miRNAs has increased due to their regulatory abilities and orchestration in both normal development and pathological conditions such as cancer [13,14].

MiRNAs are a class of small non-coding RNA that are involved in the regulation of various cellular processes, including those in tumor cells, by directly destroying its intracellular messenger RNA (mRNA) target or inhibiting the translation of its targeted mRNA, due to the complementarity between the miRNA and its target [15,16]. The miRNA-induced silencing complex (miRISC) binds to reverse complementary sequences within the 3’-untranslated region (UTR) of target mRNAs. MiRNA binding inhibits the translation and can promote the degradation of mRNA targets that mediate translational repression, deadenylation or decapping of target transcripts. RISC-bound mRNA can be localized to P-bodies, where they are stored or degraded [17,18,19]. MiRNAs can also shuttle between the cytoplasm and the nucleus. Shuttling of miRNAs is controlled by various mechanisms, which largely depend on cell type and cellular state. The non-canonical activities of miRNAs in the nucleus include transcriptional gene activation and transcriptional gene silencing [19].

Thus, miRNAs can influence the occurrence, progression, and metastasis of GBM. In the literature, there is already scattered information about the miRNAs signatures and its levels in the context of the development and further prognosis of GBM [20]. Prognostic signatures of miRNAs have been identified for certain miRNAs that are statistically significantly associated with different grades of malignancy [21]. For example, protective miRNA, miR-519a, targeted STAT3/Bcl2 signaling pathway, increased chemosensitivity, and promoted autophagy [22]. Furthermore, vice versa risk-associated miR-133a promotes tumor necrosis factor-related apoptosis-inducing ligand (TRAIL) resistance by suppressing DR5 expression and activating NF-kB signaling [23]. In addition, it is worth highlighting a group of diagnostic miRNAs, the expression level of which may differ in tumor tissue when compared with healthy brain tissue. For example, the level of miR-1271 which targets Bcl-2 was decreased in patients with GBM [24]. Recently, miRNAs were considered as therapeutic agents in the development of new drugs against glioblastoma [21,25]. It has been shown that miR-10b is a promising candidate for the development of new therapies against GBM, since the modulation of its function affects the cell cycle and the regulation of splicing in CSCs and reduces the growth of intracranial GBM xenografts [26].

In this work, we determined the miRNA and mRNA signatures of immortalized U87 MG and patient-derived glioblastoma BR3 that are differentially expressed during the transition from adherent to neurosphere cell cultures. Comparing patterns of up/downregulated miRNAs with sets of their down/upregulated mRNAs-targets we described the number of miRNA/mRNA axis that could contribute to the regulation of GBM NS formation.

Since neurospheres are a relevant cellular model for studying cancer stem cells, the results of this work can be used to form therapeutic signatures in the development of targeted drugs for the treatment of GBM.

## 2. Materials and Methods

### 2.1. Cell Lines

Human U87 MG cell line was obtained from the cell culture collection of the Institute of Molecular and Cellular Biology of the SB RAS (Novosibirsk, Russia). The cells were cultivated in Minimum Essential Medium α (MEM α; Sigma-Aldrich, St. Louis, MO, USA) with 10% FBS (Gibco BRL Co., Gaithersburg, MD, USA), 2 mM L-glutamine (Sigma-Aldrich, USA), 250 mg/mL amphotericin B, and 100 U/mL penicillin/streptomycin (Gibco BRL Co., Gaithersburg, MD, USA) at 37 °C in a humidified atmosphere containing 5% CO_2_.

### 2.2. Patient-Derived Cell Culture

Cancer tissue sample was obtained with informed consent from patient at the Novosibirsk Research Institute of Traumatology and Orthopedics n.a. Ya.L. Tsivyan (Novosibirsk, Russia). The study was approved by the Committee on the Ethics of Novosibirsk Research Institute of Traumatology and Orthopedics n.a. Ya.L. Tsivyan (protocol number No. 050/17 68 of 11 September 2017).

Glioblastoma tissue specimen was mechanically dissociated in Iscove’s modified Dulbecco’s media (IMDM, Sigma-Aldrich, St. Louis, MO, USA). Specimen dissociated into single cells was washed with 10× excess by volume of phosphate-buffered saline (PBS) and separated cells were collected by centrifugation at 300× *g*. Cells were plated in IMDM medium with 10% FBS, 2 mM L-glutamine, 100 U/mL penicillin, 100 μg/mL streptomycin, and 250 mg/mL amphotericin B for cell adhesion. The sample was given the name BR3. At the next passages, cells were cultured in complete IMDM medium supplemented with Mito + Serum Extender (BD Biosciences—Discovery Labware, San Jose, CA, USA), 2 mM L-glutamine, 100 U/mL penicillin, 100 μg/mL streptomycin, 250 mg/mL amphotericin B and were cultivated in 6-well plates at 37 °C in a humidified atmosphere containing 5% CO_2_. When 70–80% confluence was reached, cells were harvested using Triple-Express (Gibco BRL Co., Gaithersburg, MD, USA) and subcultured for further experiments. The analyzed BR3 glioblastoma culture does not have mutations in the coding regions of the IDH1 and IDH2 mRNAs. The cell culture was tested negative for mycoplasma contamination.

### 2.3. Cell Culture for Neurosphere Formation

For neurospheres formation U87 MG and BR3 cells were cultured in Dulbecco’s Modified Eagle Medium: Nutrient Mixture F-12 (DMEM: F12, Sigma-Aldrich, USA) supplemented with B-27 and N-2 Supplements, 20 ng/mL bFGF (Gibco BRL Co., Gaithersburg, MD, USA) and 20 ng/mL EGF (Sigma-Aldrich, USA) in non-treated cell culture dishes (Eppendorf, Germany) at 37 °C in a humidified atmosphere containing 5% CO_2_. There were 3–4 passages for each culture. Phase-contrast microscopy was performed using the Nicon Eclipse Ti-S microscope (Nikon, Japan).

### 2.4. RNA Isolation

Total RNA and small RNA (<200 nucleotide length) fractions were extracted from cells with LRU RNA extraction kit (Biolabmix, Russia) following the manufacturer’s protocol. RNA concentration was assessed using Qubit RNA HS Assay Kit (Thermo Fisher Scientific, Waltham, MA, USA) with Qubit 2 fluorometer (Thermo Fisher Scientific, USA). The quality of total RNA expressed as RNA Integrity Number (RIN) was determined with Bioanalyzer 2100 instrument (Agilent, Santa Clara, CA, USA) using Agilent RNA Pico 6000 Kit (Agilent, USA). The threshold RIN reading greater than 8.0 was taken as cut-off point for transition to the stage of cDNA library preparation.

### 2.5. RNA Sequencing

The construction of cDNA libraries was performed according to a standard protocol using a NEBNext Multiplex Small RNA Library Prep Kit for Illumina (New England Biolabs, UK) for small RNA fraction, NEBNext Ultra II Directional RNA library preparation kit for Illumina (New England Biolabs, Hitchin, UK) and NEBNext mRNA Magnetic Isolation Module (New England Biolabs, UK) for poly(A)+ RNA fraction. For the prepared sequencing libraries, fragment size distribution was analysed using a Bioanalyzer 2100 instrument (Agilent, USA) with an Agilent High Sensitivity DNA Kit (Agilent, USA) and quantification by Qubit DNA HS Assay Kit (Thermo Fisher Scientific, USA) with Qubit 2 fluorometer (Thermo Fisher Scientific, USA). Fragment size ranges between 100 bp to 200 bp and between 250 bp to 700 bp were observed for small RNA and poly(A)+ RNA libraries respectively. Libraries were sequenced on Illumina NextSeq 1500 instrument in 100-base-pair-single-end mode (NextSeq 500/550 High Output v2.5 Kit (Illumina, USA)). The construction of cDNA libraries and massive parallel sequencing were conducted at the Institute of Fundamental Medicine and Biology, Kazan Federal University (Kazan, Russia).

### 2.6. Transcriptome Analysis

Raw sequencing reads (100-nucleotide single-end reads) were subjected to Illumina adapter removal by Trimmomatic [27]. Adapter trimmed sequencing reads were filtered with Bowtie2 [28] using reference, containing sequences of human: rRNAs (RefSeq); tRNAs; snRNA; SINE-, LINE-, DNA-repeats consensus sequences (RepBase [29]); low complexity simple repeats, as well as mitochondrial DNA (NC_012920.1). Filtered reads were mapped to human genome (GRCh37/hg19) with STAR 2.7.1a [30] using RefGene human genome annotation (https://hgdownload.cse.ucsc.edu/goldenPath/hg19/database/, accessed on 5 June 2021). Aligned reads were quantified with QoRTs v1.3.6 [31]. For the quantification of mature miRNA sequencing reads miRBase v20 genome annotations for GRCh37/hg19 were used [32]. Differential gene expression analysis performed with DESeq2 1.36.0 [33], R version 4.1.3 and Bioconductor 3.14. The results of differential gene expression analysis—lists of up/down regulated genes and mature miRNAs were analyzed with Enrichr using R interface [34]. For the analysis of relationships of miRNAs and mRNA-targets we used both miRNet [35] and Enrichr [34].

## 3. Results

### 3.1. Neurospheres Formation from Primary Brain Tumor BR3 and Immortalized Cell Line U87MG

In order to obtain patient-derived glioblastoma cells BR3 we used solid primary brain tumor obtained from treatment-naive patient. BR3 primary brain tumor was acutely dissociated into individual cells. We used culture conditions that favored stem cell growth, developed for the isolation of neural stem cells in the form of neurospheres [36,37,38]. We also cultivated immortalized U87 MG cells in the same conditions for neurospheres formation (Table 1). U87 MG was collected at 3 passages. BR3 was collected at 4 passages.

**Figure 1:**
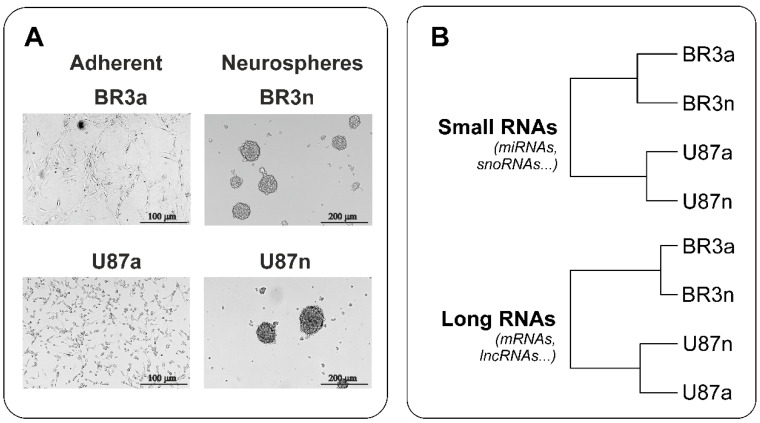
(**A**) Representative images of MN (“a”, adherent) and NS (“n”, neurospheres) glioblastoma cells before cell collection for NGS RNAseq analysis. (**B**) Euclidean distance trees of glioblastoma cell cultures constructed from gene expression data subjected to variation stabilizing transformation (VST). The distance trees for the small RNAs and long RNAs cDNA-libraries are shown separately. The complete agglomeration method for clustering was used.

Both patient-derived BR3 and immortalized U87 MG glioblastoma cells formed neurospheres (Figure 1A). The efficiency of neurospheres formation by U87 MG cells was higher; they reached a size of 150 μm in 3–4 days, while the size of neurospheres formed by BR3 cells reached of 150 μL in 4–5 days. At the same time, under monolayer conditions in the presence of serum in the medium, the cells of both cultures adhered to the culture dishes and acquired a stellate morphology typical of glial cells (Figure 1A).

### 3.2. Transcriptome and microRNome Changes of Neurospheres Occurs in Common Way in Patient-Derived and Immortalized Glioblastoma Cell Cultures

We performed NGS-analysis based on Illumina 1500 platform both of long RNAs and small RNAs and obtained from ~9.4 × 10^6^ to ~4.6 × 10^7^ experimental reads for each of two GBM cell cultures in adherent and neurosphere culture conditions (Table 1).

We aligned the primary sequencing data of long and small RNA cDNA-libraries with the human genome (GRCh37/hg19), and performed a differential analysis of GBM neurospheres gene expression with the corresponding monolayer cultures.

With hierarchical clustering (HC) of RNA sequencing data it was determined that patient-derived BR3 RNA-patterns gathered in a separate branches, clearly distinguished from U87 MG (Figure 1B). Furthermore, it can be expected that changes in the transcriptomes of BR3 and U87 MG cells under conditions of NS formation are characterized by common specific short and long transcripts, such as common miRNAs and common mRNAs.

**Figure 2:**
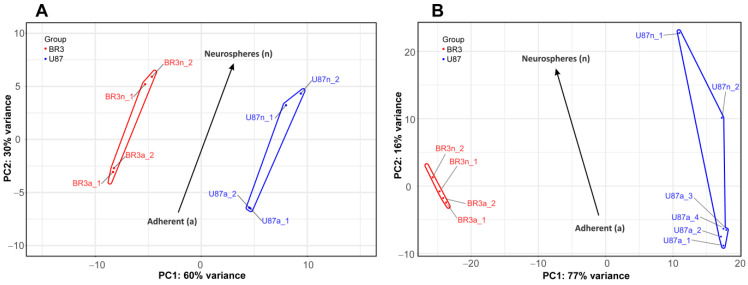
Principal component analysis (PCA) of DESeq2 normalized, variance stabilizing transformed (VST) gene expression data. PC1:PC2 graphs for small RNAs (**A**), and long RNAs (**B**) NGS-libraries. Cell culture specific PC1:PC2 points are annotated with colored envelopes. The black arrows show the general trends of PC1:PC2 transition from the MN (“a”, adherent) to the NS state (“n”, neurospheres).

Consistent with this, PCA shows that the small and long RNA patterns of U87 MG a/n cells form distinct non-overlapping regions of PC1:PC2 dots with those in patient-derived BR3 a/n cultures (Figure 2). However, a general direction of PC1:PC2 changes can be identified for both cell cultures, for both small and long RNA sets (Figure 2A,B). This indicates that transcriptome changes in the process of glioblastoma NS formation are determined to a greater extent by the initial cell-specific context of gene expression, but strongly modulated by the conditions of cultivation in the presence of bFGF and EGF.

### 3.3. Common miRNA and mRNA Expression Changes in Both Patient-Derived BR3 and Immortalized U87 MG GBM Cell Cultures

**Figure 3:**
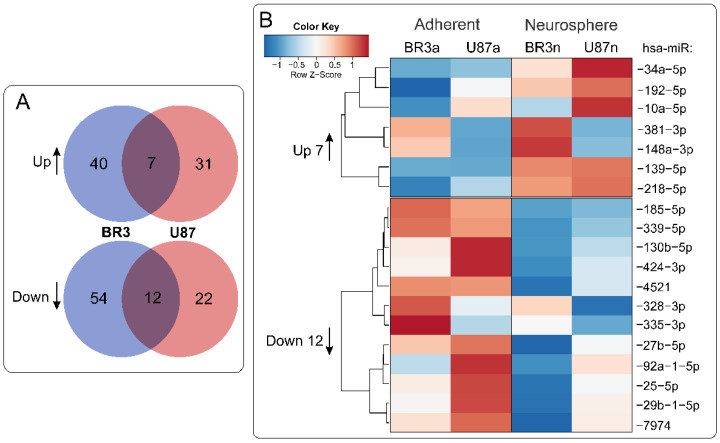
(**A**) Venn diagrams showing intersections of miRNA sets of BR3 and U87 GBM cultures, separately for miRNAs with increased (Up) or decreased (Down) levels of NS compared to the corresponding adherent cultures. (**B**) Heatmap of 7 commonly upregulated and 12 commonly downregulated miRNAs in NS vs. MN GBM cells.

When we compared lists of differentially expressed miRNAs it was determined that 7 miRNAs were upregulated and 12 were downregulated commonly and unidirectionally, both in BR3 and U87 MG cells under conditions of NS formation (Figure 3).

**Figure 4:**
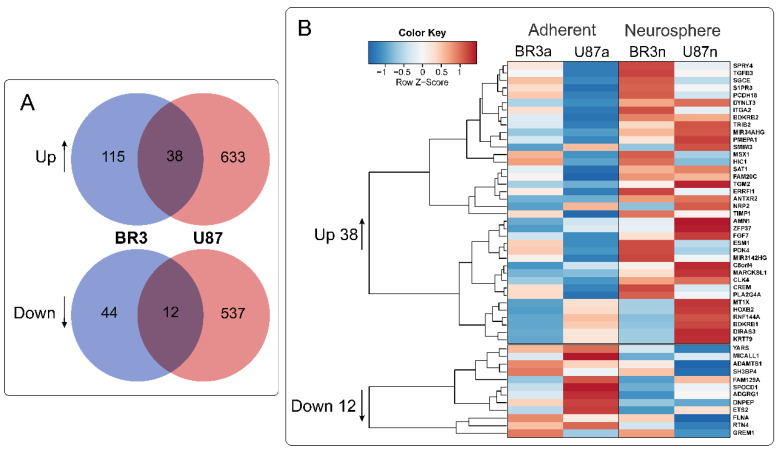
(**A**) Venn diagrams showing intersections of gene sets of BR3 and U87 MG GBM cells, separately for genes with increased (Up) and decreased (Down) levels for NS cultures compared to the corresponding MN. (**B**) Heatmap of 38 commonly upregulated and 12 commonly downregulated genes in NS vs. MN glioblastoma cells.

36 mRNAs (and two lncRNAs) were commonly and unidirectional upregulated while 12 mRNAs were downregulated in both of BR3 and U87 MG neurospheres (Figure 4). Interestingly, two upregulated lncRNAs (MIR34AHG and MIR3142HG) represented by the products of miRNA host-genes. An increase in the level of lncRNA MIR34AHG exons is accompanied by an increase in the miR-34a-5p miRNA level (Figure 3A). Thus, it can be proposed that the upregulation both of lncRNA MIR34AHG and miR-34a can be associated with the activation of the gene transcription, rather than with post-transcriptional regulation of pre-lncRNA MIR34AHG maturation.

In order to outline the main cellular processes in which the products of these mRNAs are involved, we used Erichr library “MSigDB_Hallmark_2020” (Table 2). In both patient-derived BR3 and immortalized U87 MG GBM cells processes of NS formation proceeded with an increase in mRNA levels related to groups of “Epithelial Mesenchymal Transition” and “KRAS Signaling Up”, “TNF-alpha Signaling via NF-kB”, “Hypoxia”, “Apoptosis”, “Angiogenesis” (Table 2, “Upregulated”). Commonly decreased mRNAs involved in “mTORC1 Signaling”, “Epithelial Mesenchymal Transition”, “Glycolysis”, “Hedgehog Signaling”, “UV Response Dn” and in “PI3K/AKT/mTOR Signaling” as well (Table 2, “Downregulated”).

### 3.4. Relationships between Changes in miRNA and mRNA Levels in GBM Cell Cultures under Conditions of Neurosphere Formation

It is known that miRNA binding inhibits translation and can reduce the level of target mRNAs, which provides a basis for inverse correlation of relative miRNA/mRNA expression levels [18,19].

We used Enrichr [34] and miRNet [35] databases to explore possible relationships between changes in miRNA and mRNA levels in GBM cell cultures under conditions of neurosphere formation (Table 3). It was determined that downregulated miR: -130b-5p, -25-5p, -335-3p, and -339-5p have mRNA targets, the level of which were upregulated: BDKRB2, SPRY4, ERRFI1 and TGM2. Upregulated miRNAs miR: -139-5p, -148a-3p, -192-5p, -218-5p, -34a-5p, and -381-3p have mRNA-targets the level of which were downregulated: RTN4, FLNA, SH3BP4, DNPEP, ETS2, MICALL1, and GREM1 (Table 3).

### 3.5. The miRNA-mRNA Network Allows to Suggest Cell Processes and Signaling Pathways Regulated by miRNAs in Glioblastoma Neurospheres

**Figure 5:**
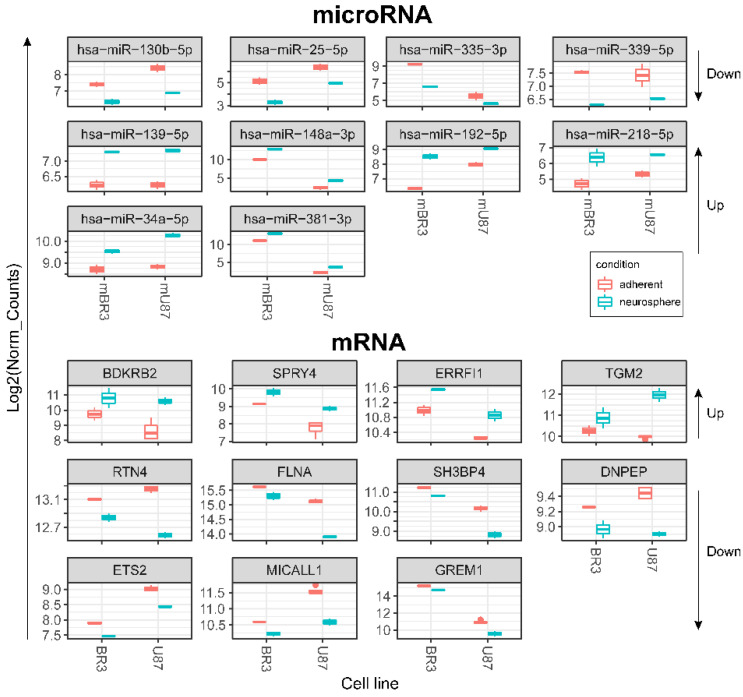
Box plots of DESeq2 normalised expression values of mRNAs and miRNAs grouped by GBM cell cultures and coloured red for monolayer (adherent) cultures and cyan for corresponding NS. Differentially expressed transcripts meet the criteria: DESeq2 pval < 0.05, Log2FoldChange > 0 for upregulated or log2FoldChange < 0 for downregulated (for both BR3 and U87 adherent/neurosphere pairs in one direction—unidirectionally “up” or “down”). Arrows on the right show direction of RNA expression changes in GBM NS for the horizontal block of plots.

We have found that neurosphere formation by BR3 and U87 MG human GBM cells accompanied by downregulation of miRNAs miR-130b-5p and miR-25-5p both of which targeted to upregulated mRNA SPRY4 (Table 3, Figure 5). It is known that SPRY4 is a factor that suppresses the FGF/FGFR—signaling by interacting with serine/threonine-protein kinase RAF1 (CRAF) and inhibiting its activity [39]. SPRY4 is considered as a tumor suppressor, its activation reduces the proliferation and migration of GBM cells [40]. Our data (Table 3) suppose that downregulation of miRNAs miR-130b-5p and miR-25-5p can stabilize targeted mRNA SPRY4 and thus provide conditions for the suppression and feedback regulation of FGF/FGFR-signaling.

Downregulation of miR-335-3p is accompanied with increased level of its target mRNA ERBB receptor feedback inhibitor 1 (ERRFI1) (Table 3, Figure 5). *ERRFI1* is considered as a tumor suppressor gene in relation to GBM [41]. *ERRFI1* gene product—MIG-6 negatively regulates EGFR enhancing its internalization and degradation and reducing EGFR tyrosine kinase activity [42]. Thus, downregulation of miR-335-3p level may promote greater stability of ERRFI1 mRNA and suppress EGFR signaling in GBM NS.

Thus, miR-130b-5p, miR-25-5p and miR-335-3p targeted to mRNAs SPRY4 and ERRFI1 can participate in feedback regulation of FGF/FGFR-signaling and provide the basis of fine tuning of GBM NS formation.

Downregulation of miR-130b-5p complemented by increased level of its target mRNA BDKRB2 (Table 3, Figure 5) which is associated with patient survival rate and more malignant glioma phenotypes. Furthermore, BDKRB2 was involved in the EMT process and is considered as a prognostic marker of glioma [43]. Thus, the miR-130b-5p/BDKRB2 axis can be considered as an integral part of the pathogenesis of glioma.

Previously it was shown that upregulation of TGM2 was significantly increased in recurrent patients with mesenchymal subtype of GBM and inversely correlated with patient prognosis. Moreover, it was shown that TGM2 is upregulated in the perinecrotic region of GBM and triggered mesenchymal transdifferentiation of glioma stem cells [44]. Our data allow to associate increased level of TGM2 mRNA with lowered level of hsa-mir-339-5p in GBM NS (Table 3, Figure 5), which indicates an important role of this miRNA in gliomagenesis.

We detected that the level of mRNA of reticulon 4 (RTN4) was downregulated in GBM cells at the process of NS formation. Wherein downregulation of RTN4 mRNA is associated with increased level of 4 miRNAs:-139-5p, -148a-3p, -218-5p, -34a-5p (Figure 5, Table 3). RTN4 has previously been shown to regulate lipid homeostasis and cytoskeletal modulation. Moreover, in a sphingolipid-dependent manner, downregulation of RTN4 led to disruption of the localization of AKT in the plasma membrane, due to which AKT phosphorylation, which is involved in many cancers, was significantly reduced [45]. RTN4 and its receptors are highly expressed in glioma tumor cells, indicating that glioma cells may promote tumor proliferation through an autocrine process [46]. Thus, the above miRNAs can be considered as potential regulators of proliferation, migration and invasion of GBM cells due to their effect on mRNA RTN4.

The decreased level of FLNA mRNA in GBM NS is complemented by an increased level of two miRNAs miR-148a-3p and miR-218-5p (Figure 5, Table 3). Filamin A (FLNA), also known as actin-binding protein 280, functions as scaffold molecule to facilitate protein–protein interactions and influence cellular protein localization. It plays a dual role because overexpression of FLNA has a tumor-promoting effect only when it is localized in the cytoplasm, whereas if FLNA is proteolyzed and the resulting C-terminal fragment is localized in the nucleus, it inhibits tumor growth and metastasis [47]. Phosphorylation of FLNA required for GBM migration and invasion can be initiated by several protein kinases, including cyclic AMP (cAMP)-dependent protein kinase, p90 ribosomal kinase, PAK1, cyclin D1/Cdk4, PKCα and mediated by mTORC2, the last one induces actin changes in the cytoskeleton and further influences cell motility and invasiveness [48].

The mRNA of SH3 Domain Binding Protein 4 (SH3BP4) was downregulated in GBM NS and it’s associated with upregulation of miR-192-5p (Figure 5, Table 3). SH3BP4 is considered as a tumor suppressor that functions in the amino acid-mTORC1 pathway. It is also suggested that due to the presence of the DD motif near the C-terminus, SH3BP4 can regulate cell death. It is worth noting that SH3BP4 gene locus is often either deleted in many human cancers, or there is a loss of heterozygosity [49].

DNPEP (aspartyl aminopeptidase) expression is frequently downregulated in breast cancer tissues and regarded as tumor suppressor in vitro and in vivo breast cancer models [50]. Our data allowed connecting downregulation of DNPEP mRNA with upregulation of hsa-miR-218-5p in the process of glioblastoma NS formation (Figure 5, Table 3).

An increase in the level of two miRNAs miR-218-5p and miR-34a-5p in GBM NS was accompanied by a decrease in the level of their target mRNA ETS2 (Figure 5, Table 3). It is known that ETS2, complexing with ΔNp73 (an N-terminal truncated isoform of TP73), increases the expression of ANGPT1 and Tie2, and thus promotes GBM angiogenesis by causing vascular sprouting [51].

MICALL1 (MICAL Like 1) regulates endocytosed-EGF receptor trafficking. Knocking down MICALL1 promotes degradation of EGFR [52]. Our data allow to associate the decrease in MICALL1 mRNA expression with an increase in the level of hsa-miR-34a-5p in GBM NS (Figure 5, Table 3). Thus, miR-34a-5p/MICALL1/EGFR axis could provide the basis for feedback regulation of GBM NS formation.

GREM1 (Gremlin-1) belongs to a family of strong secreted BMP antagonists such as Cerberus and Dan subfamilies. In gliomas Gremlin-1 blocks prodifferentiation effects of BMPs, and overexpression of Gremlin-1 in non-CSCs decreases their endogenous BMP signaling to promote stem-like features thus maintaining glioblastoma tumor proliferation and glioblastoma hierarchies [53]. In cells of BR3n and U87n cultures the level of miRNA miR-381-3p was increased in accordance with the reduced level of their target mRNA GREM1 (Figure 5, Table 3). Thus, the miR-381-3p/GREM1 axis may reflect the processes of GBM intercellular communication in the formation of NS.

**Figure 6:**
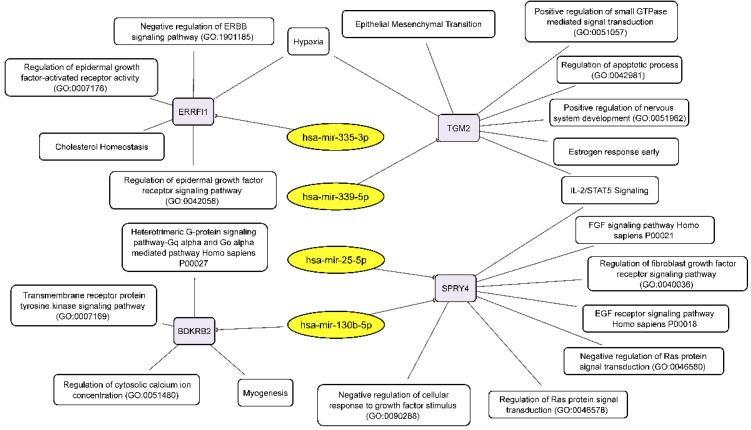
Integrated network analysis of downregulated miRNAs and their activated target mRNAs in GBM NS, as well as the participation of the latter in cellular processes. Yellow ovals represent miRNAs, gray round rectangles—mRNA-targets, white round rectangles—cellular processes and signaling pathways.

Combining our RNA-seq data on the relationship between miRNAs and their target mRNAs, we can draw up two schemes linking the discussed RNAs to the processes that govern the formation of neurospheres by glioblastoma cells. Downregulated miRNAs, through their target mRNAs, influence the following key cellular processes: EMT, negative regulation of ERBB signaling pathway, regulation of epidermal growth factor activated receptor activity, negative regulation of cellular response to growth factor stimulus, negative regulation of Ras protein signal transduction, regulation of apoptotic process and others (Figure 6).

**Figure 7:**
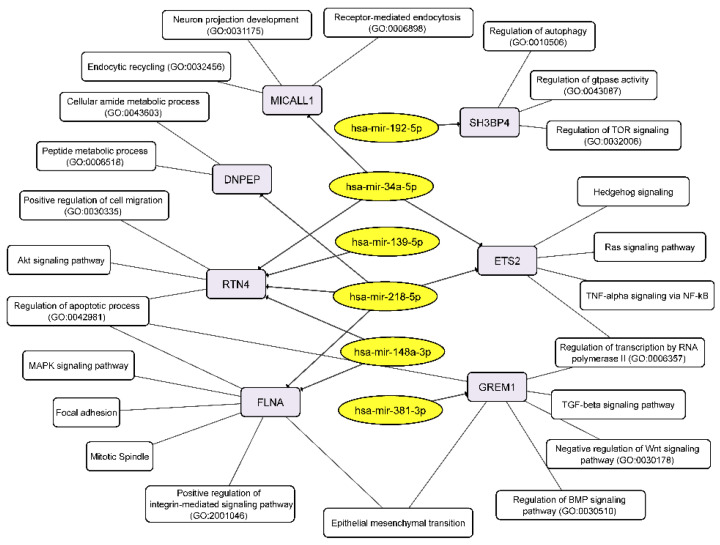
Integrated network analysis of the GBM NS upregulated miRNAs and their targeted downregulated mRNAs, as well as the participation of the latter in cellular processes. Yellow ovals represent miRNAs, gray round rectangles—mRNA-targets, white round rectangles—cellular processes and signaling pathways.

Conversely, upregulated miRNAs control the following cellular processes via their target mRNAs: receptor-mediated endocytosis, endocytic recycling, peptide metabolic process, positive regulation of cell migration, apoptotic process, MAPK signaling, mitotic spindle, regulation of BMP signaling pathway, negative regulation of WNT signaling pathway, and others including EMT (Figure 7).

### 3.6. In Silico Validation of the Relationship between miRNA and mRNA Expression in Glioblastoma Cells with Data from the TCGA Project

In order to estimate the correlation between levels of miRNA and their mRNA targets in GBM cells, we used data of the Cancer Genome Atlas Program (TCGA) as an independent approach. For this, data on the expression of miRNAs, mRNAs from the Glioblastoma Bio Discovery Portal (GBM-BioDP) were used [54] (Supplementary Tables S1–S3). When analyzing the data in each series miRNA/mRNA pairs, we excluded only one outlier point according to the interquartile range approach.

Inverse correlation of relative expression levels was observed for 9 miRNA/mRNA pairs using the generally accepted classification of GBM into molecular subtypes: classical (C), neural (N), mesenchymal (M) and proneural (P) as well as unknown GBM subtype (U) according to the data of GBM-BioDP [54,55] (Table 4, and Figure S1). With that, the Pearson correlation coefficient (R^2^) for a linear approximation of the average levels mRNA/miRNA expression ranged from 0.51 to 0.98.

For the pairs: FLNA/miR-148; MICALL1/miR-34a; RTN4/miR-139; RTN4/miR-218; and TGM2/miR-339 no inverse correlations with Pearson’s R^2^ > 0.5 were observed. It should be noted that in the GBM-BioDP database there are no data on the expression levels of miR-130b-5p (miR-130b*) and miR-25-5p (miR-25*) (Tables S1 and S2). Therefore, we could not analyze the relationship between expression levels in miRNA/mRNA pairs: BDKRB2/miR-130b*; SPRY4/miR-130b*; and SPRY4/miR-25*.

Table 4 shows that DNPEP and ERRFI1 mRNAs levels are predictive of overall survival of patients with mesenchymal and proneural GBM subtypes, respectively. The levels of miRNAs miR-34a and -148a can be considered as potential predictors of survival in patients with proneural and neural GBM subtypes.

Thus, in general, our data on oppositely directed changes in the levels of miRNAs and their mRNA-targets find confirmations in the transcriptomic data of the TCGA project for GBM tissues and cell cultures. In addition, the findings highlight that the microRNA/mRNA axes identified during the formation of neurospheres in culture may be associated with fundamental processes affecting tumor growth and invasion and, ultimately, patient survival.

## 4. Discussion

Glioblastoma is the most aggressive brain tumor, characterized by a low 5-year survival rate, as well as high invasiveness in healthy brain tissues. Due to the insufficient effectiveness of the available therapy for glioblastomas and the need to develop new targeted drugs, the role of relevant cell models for the search and development of such drugs has increased. At the same time, the cell models themselves can be enriched with tumor stem cells, which make such a study even more valuable [7].

Glioblastoma, like any cancer, is a pathological condition in which gene expression is dramatically impaired. A large number of reviews have been written on the differential expression of miRNAs in human glioblastoma cells and in the tumor itself. MicroRNA belongs to the family of small non-coding RNAs and has a size of about 18–25 nucleotides; its maturation up to the formation of a protein complex termed RNA-induced silencing complex (RISC) is a multistage process in the nucleus and cytoplasm [13,14]. The mature miRNA is incorporated into RISC and guides it to target mRNA. MiRNA binding inhibits the translation and can promote the degradation of mRNA targets that mediate translational repression, deadenylation or decapping of target transcripts [17,18,19]. Thus, miRNAs can not only suppress translation, but also can potentially reduce the level of target mRNAs in cells. A change in the level of an individual microRNA and its target mRNA in opposite directions can indicate the participation of this miRNA in the regulation of target gene expression. Due to their direct and indirect effects on target mRNAs, miRNAs are attracting more and more attention both in terms of the development of predictive markers of glioblastoma and in terms of the development of targeted drugs [13].

However, currently there are few studies analyzing miRNAome and whole transcriptome, describing relationships of miRNAs and mRNA-targets in glioblastoma tumor tissue or in cell cultures [56,57,58]. In one of the first integrated analysis of miRNA and mRNA expression revealing the mechanism of tumor initiation and progression: miRNA coexpression network was constructed and 19 important microRNAs were found, 3 of which were significantly associated with the survival of patients with glioblastoma [56]. A detailed analysis of the small RNA transcriptome of the U87MG cell line, a grade IV glioma cell line, and its changes under hypoxic conditions is presented for the first time [57]. Using NanoString miRNA expression analysis and PCA miRNA expression profiles were determined in 13 frozen brain tissue samples (9 GBM and 4 controls). The most highly expressed miRNA was miR-21. MiR-138 was identified as one of the miRNAs with reduced expression in GBM samples [58].

Recently Tomei et al. [59] studied differences in miRNA-content between human GBM of autologous CSCs and differentiated tumor cells obtained from the same GBM patients. MiR-21 and miR-95 were among the most significantly deregulated miRNAs, and their expression was also associated with patient survival [59]. MiRNA-21-5p (miR-21) is considered the most frequently upregulated miRNA in various types of cancer. The NANOG/Stat-3 signaling pathway has been found to play a key role in miR-21 production [60,61]. Downregulation of miR-95 has been shown to affect glioma cell proliferation, invasion and apoptosis by targeting CELF2. Furthermore, it was shown that miR-95 expression levels are positively associated with glioma grade [62]. In our study, we did not find reliable correlations between changes in the level of these miRNAs and sphere formation.

Due to the frequent use of neurospheres as relevant models in studies of glioblastoma, the aim of this study was to determine miRNA and mRNA signatures that contribute to the neurospheres formation. To achieve this goal, it was necessary to perform the transcriptome and miRNome analysis of the immortalized and primary glioblastoma cell cultures in adherent and neurosphere-forming conditions, for analyzing this data using bioinformatic tools.

According to PCA analysis the transcriptome and miRNome changes of neurospheres occurred with common features in patient-derived BR3 and immortalized glioblastoma U87 MG cell cultures. NS formation is characterized by seven differently expressed unidirectionally upregulated and 12 downregulated miRNAs (Figure 3). For these 19 differentially expressed miRNAs, we identified four downregulated miRNAs with four upregulated target mRNAs and six upregulated miRNAs with seven corresponding downregulated mRNA-targets (Table 3 and Figure 5).

The observed downregulated miRNAs (Table 3, Figure 5 and Figure 6) have previously been described in experimental studies of various diseases and malignancies, including gliomas. It is known, that the level of miR-130b-5p was increased in glioma tissues and cell cultures, and its upregulated expression promoted the proliferation and invasion of glioma cells, inhibited apoptosis of the cells in vitro, and induced their tumorigenicity in vivo [63,64]. Downregulation of miR-130b-5p observed in patients with coronary artery disease (CAD), with negative correlation with SYNTAX score and stenosis in female CAD patients [65]. It was observed that miR-25-5p is involved in the pathogenesis and processes of vascular diseases by targeting neuronal growth regulator 1 (NEGR1) via regulating the JAK/STAT signaling pathway [66]. Several studies showed the involvement of this miRNA in endothelial cell proliferation, migration and apoptosis in CAD [67]. MiR-335-3p inhibition by upregulated lncRNA CASC9 promoted proliferation, migration, and invasion of non-small cell lung cancer [68]. MiR-339-5p is a tumor suppressor, inhibiting PTP4A1/HMGB1 signal pathway it suppresses vasculogenic mimicry, migration, and invasion of brain glioma U251 cells [69]. Downregulation of miR-339-5p was recently revealed in glioma [70], where overexpressed of miR-339-5p has been shown to inhibit the proliferation, invasion and migration of U87 MG human GBM cells and promote programmed cell death.

The group of commonly upregulated 6 miRNAs (Table 3, Figure 5 and Figure 7) is also known in cancer studies, including glioma. It has been shown that overexpression of miR-139-5p in vitro leads to the inhibition of cell proliferation, migration and invasion of glioma by targeting gamma-aminobutyric acid A receptor alpha 1 (GABRA1). Moreover, a correlation was found between the level of this miRNA, the probability of survival and the World Health Organization grade [71]. MiR-148a-3p is considered to be a tumor suppressor that is significantly reduced in several types of cancer, such as ovarian cancer, and gastric cancer [72,73]. Overexpression of miR-148a/b-3p inhibited VEGF-induced activation of VEGFR2 and its downstream pathways by regulating expression of neuropilin-1 (NRP1) [74]. Various studies have identified that miR-192-5p has several target mRNAs, for example, p53 and a regulatory gene that targets the p53 signaling pathway, BIM, RB1, SEMA3A, therefore, it was suggested that it is a tumor suppressor in various malignant carcinomas [75]. MiR-218-5p can specifically bind to lipoma HMGIC fusion partner-like 3 (LHFPL3) mRNA, resulting in inhibition of EMT, decreased cellular activity, proliferation, and invasive capacity [76]. MiR-34a (hsa-mir-34a-5p) targets many oncogenes associated with proliferation, apoptosis, and glioma invasion and so have been characterized as a tumor suppressor. Moreover, it is proposed as a predictive miRNA, lower level of which associated with worse physical functioning, higher tumor volume, greater depressive symptom severity of GBM patients and the lower Karnofsky performance index [77]. Several studies have shown the suppressive role of miR-381 (hsa-miR-381-3p) in cell metastasis and EMT in glioblastoma due to the suppression of LEF1 expression. In another study this miRNA inhibited malignant behavior of glioma cells by targeting ANTXR1 [78].

In total, for ten differentially expressed miRNAs we found 4 upregulated: BDKRB2, SPRY4, ERRFI1, TGM2 and seven downregulated mRNA-targets: RTN4; FLNA, SH3BP4, DNPEP, ETS2, MICALL1, GREM1 (Table 3 and Figure 5). Published data on differentially expressed microRNAs and their target mRNAs make it possible to reveal cellular processes and signaling cascades regulated by microRNAs in neurospheres. (Figure 6 and Figure 7). There is a fine regulation of GBM cell proliferation, since we see, that the upregulation of the BDKRB2 and TGM2 mRNAs (Figure 5) leads to stimulation of the EMT, which is necessary for tumor progression migration and metastasis [43,44]. There was mRNAs downregulation of tumor suppressors such as SH3BP4 and DNPEP (Figure 5), which influence oncogenesis via the amino acid-induced TOR signaling and PAK5–DNPEP–USP4 pathway, respectively [49,50]. Downregulated mRNAs (RTN4, FLNA, GREM1) also indicate the fine tuning of the neurosphere formation. RTN4, which promotes tumor proliferation in glioma via Akt signaling pathway, is downregulated in GBM NS [45,46]. GREM1 suppression impairs growth and self-renewal [53]. During NS formation the reduced level of ETS2 mRNA was also observed (Figure 5), which may indicate a lack of focus on angiogenesis either, since it is a reverse post-invasive process that requires the EMT [51].

Moreover, during neurospheres formation, changes in four miRNAs (hsa-miR-130b-5p, hsa-miR-25-5p, hsa-mir-335-3p, hsa-miR-34a-5p) at once through their mRNA targets (SPRY4, ERRFI1, MICALL1) negatively affect the signaling cascades of EGFR and FGFR, respectively. The mRNAs SPRY4 and ERRFI1 participate in feedback regulation of FGFR-signaling by interacting and inhibiting CRAF or EGFR kinase, respectively [39,40,41,42]. In its turn, MICALL1 inhibits tumor growth through EGFR degradation [52]. Thus, the involvement of SPRY4, ERRFI1, and MICALL1 in the regulation of EGFR/FGFR signaling feedback emphasizes the important role of hsa-miR: -130b-5p, -25-5p, -335-3p, and -34a-5p not only in the formation of NS, but also in the regulation of malignant growth and invasion of GBM through a negative feedback loop.

## 5. Conclusions

In this work, we determined the miRNAs that are differentially expressed and inversely correlated with mRNAs in the process of GBM neurosphere formation. The number of miRNA/mRNA axes that may contribute to the regulation of GBM neurosphere formation were described. In general, the functional interpretation showed that during neurosphere formation miRNAs provide fine tuning of signals that regulate proliferation and differentiation with a tendency to epithelial-mesenchymal transition.

Understanding the signatures of miRNA/mRNA-axis can be useful both for the development of early diagnosis of glioblastoma and for the development of new drugs directed against the formation of cancer stem cells, and therefore against formation and invasion of glioblastoma. In the future, the questions arise: which transcription factors contribute to the regulation of the expression of mRNA and miRNA genes; why, in fact, their expression changes; and how this process can be influenced.

## Figures and Tables

**Table 1:** Characteristics of human GBM cell cultures and RNA sequencing data.

Cell Culture	Histological Characteristic	Culture Conditions *	NGS-Library **	Number of Replicates	Number of NGS-Sequencing Reads (10^6^)
BR3	GBM	MN	BR3a	2	19.55
		MN	mBR3a	2	20.23
		NS	BR3n	2	19.58
		NS	mBR3n	2	9.42
U87 ***	GBM	MN	U87a	4	46.35
		MN	mU87a	2	23.08
		NS	U87n	2	22.95
		NS	mU87n	2	22.64

* MN—monolayer (adherent), NS—neurospheres. ** The names of the NGS libraries used in this article correspond to the names of the cell cultures. Prefix “m” denotes the small RNA cDNA libraries and sequencing data. *** Furthermore, known as U87 MG human glioblastoma cell line.

**Table 2:** Cellular processes and signaling pathways. Common for differentially expressed mRNAs of U87 MG and BR3 NS cellular processes and signaling pathways determined using Enrichr. Enrichr records (library “MSigDB Hallmark 2020”), ordered by ascending *p* value of BR3.

Term	BR3		U87 MG		Common Genes *
	*p* Value	Adj. *p* Value	*p* Value	Adj. *p* Value	
**Upregulated**					
EMT **	4.63 × 10^−8^	1.57 × 10^−6^	3.62 × 10^−9^	5.70 × 10^−8^	ITGA2; PMEPA1; SAT1; TIMP1; MSX1; TGM2
KRAS Signaling Up	1.56 × 10^−4^	2.65 × 10^−3^	6.88 × 10^−8^	4.82 × 10^−7^	ITGA2; TRIB2
TNF-alpha Signaling via NF-kB	4.44 × 10^−3^	3.02 × 10^−2^	1.14 × 10^−24^	5.59 × 10^−23^	PMEPA1; SAT1
Hypoxia	4.44 × 10^−3^	3.02 × 10^−2^	1.61 × 10^−8^	1.58 × 10^−7^	TGM2; ERRFI1; TGFB3
Apoptosis	8.00 × 10^−3^	3.89 × 10^−2^	4.66 × 10^−9^	5.70 × 10^−8^	SAT1; TIMP1
Angiogenesis	3.09 × 10^−2^	1.17 × 10^−1^ ***	1.95 × 10^−7^	1.19 × 10^−6^	TIMP1; MSX1
**Downregulated**					
mTORC1 Signaling	1.99 × 10^−5^	2.19 × 10^−4^	9.43 × 10^−3^	4.93 × 10^−2^	--
EMT **	2.37 × 10^−3^	1.31 × 10^−2^	1.09 × 10^−5^	3.21 × 10^−4^	GREM1; FLNA
Glycolysis	2.37 × 10^−3^	1.31 × 10^−2^	9.43 × 10^−3^	4.93 × 10^−2^	--
HedgehogSignaling	4.56 × 10^−3^	2.01 × 10^−2^	1.65 × 10^−2^	7.76 × 10^−2^	ADGRG1; ETS2
UV Response Dn	7.67 × 10^−3^	2.81 × 10^−2^	6.47 × 10^−3^	4.93 × 10^−2^	--
PI3K/AKT/mTOR Signaling	3.50 × 10^−2^	1.10 × 10^−1^ ***	2.41 × 10^−3^	2.27 × 10^−2^	--

*—Up- or downregulated transcripts commonly detected both in BR3 or U87 MG neurospheres. **—EMT—Epithelial Mesenchymal Transition. ***—Adjusted *p* value > 0.05.

**Table 3:** Relationships between differentially expressed common mRNAs and miRNAs of BR3 and U87 MG GBM neurospheres. MicroRNA/mRNA pairs are presented, the levels of which changed in the process of NS formation in opposite directions.

miRNA	Enrichr		miRNet	
	mRNA-Targets	Library *	mRNA-Targets	Literature **
**Downregulated miRNAs—Upregulated mRNAs** ***				
hsa-miR-130b-5p	BDKRB2	miRTarBase_2017	BDKRB2; SPRY4	19536157, tarbase
hsa-miR-25-5p	SPRY4	miRTarBase_2017	SPRY4	26701625
hsa-mir-335-3p	--	--	ERRFI1	tarbase
hsa-mir-339-5p	--	--	TGM2	tarbase
**Upregulated miRNAs—Downregulated mRNAs** ***				
hsa-mir-139-5p	--	--	RTN4	tarbase
hsa-mir-148a-3p	--	--	FLNA; RTN4	tarbase, tarbase
hsa-miR-192-5p	SH3BP4	miRTarBase_2017	SH3BP4	19074876
hsa-miR-218-5p	DNPEP; ETS2; FLNA	miRTarBase_2017	DNPEP; ETS2; FLNA; RTN4	23212916; 20371350; 23212916; tarbase
hsa-miR-34a-5p	RTN4	miRTarBase_2017	ETS2; MICALL1; RTN4	tarbase; tarbase; 21566225|20371350
hsa-miR-381-3p	GREM1	miRTarBase_2017	GREM1	23824327

*—Erichr libraries. We used both “miRTarBase_2017” and “TargetScan miRNA 2017” libraries, but only the first one showed matches with the experimental sets of common (overlapping) miRNAs. **—Literature column of miRNet database containing references to external databases and PMID. ***—Shows sets of overexpressing common miRNAs and their underexpressed target mRNAs, and vice versa.

**Table 4:** Inverse correlation of miRNA levels and their proposed mRNA targets in GBM and the significance of mRNAs and miRNAs for the patient overall survival prediction according to the data of TCGA project (GBM-BioDP). C, N, M and P denotes classical, neural, mesenchymal and proneural GBM subtypes, respectively (according Verhaak et al. [55]). U—data of unknown GBM subtype according to GBM-BioDP [54]. R^2^—square of Pearson’s correlation coefficient of the linear approximation of mRNA/miRNA mean expression levels. Data detailed in Figure S1, Tables S1–S3.

mRNA		miRNA		Pearson’s R^2^	GBM Subtypes *	Kaplan–Meier Survival Curve 1Half vs. 2Half Comparison p-val							
						mRNA				miRNA			
						C	M	P	N	C	M	P	N
DNPEP	Down	miR-218	Up	0.78	C,M,N,U	0.220	0 **	0.198	0.396	0.617	0.614	0.406	0.768
ERRFI1	Up	miR-335	Down	0.75	C,M,N,U	0.673	0.063	0.222	**0.026**	0.405	0.825	0.674	0.268
ETS2	Down	miR-218	Up	0.51	M,N,P,U	0.626	0.251	0.619	0.239	0.617	0.614	0.406	0.768
ETS2	Down	miR-34a	Up	0.51	M,N,P,U	0.626	0.251	0.619	0.239	0.982	0.972	**0**	0.333
FLNA	Down	miR-218	Up	0.91	C,N,P,U	0.353	0.241	0.343	0.130	0.617	0.614	0.406	0.768
GREM	Down	miR-381	Up	0.97	M,N,P,U	0.576	0.297	0.057	0.969	0.362	0.865	0.723	0.181
RTN4	Down	miR-148a	Up	0.98	M,N,P,U	0.226	0.817	0.452	0.369	0.442	0.097	**0**	**0.014**
RTN4	Down	miR-34a	Up	0.73	C,M,N,P,U	0.226	0.817	0.452	0.369	0.982	0.972	**0**	0.333
SH3BP4	Down	miR-192	Up	0.65	C,M,N,U	0.460	0.926	0.588	0.737	0.986	0.446	0.900	0.425

*—Glioblastoma subtypes for which an inverse correlation of miRNA/mRNA levels was found. **—Kaplan–Meier survival *p*-value < 0.05 are highlighted in bold.

## Data Availability

The data presented in this study are available in this article.

## Supplementary Materials

The following supporting information can be downloaded at: https://www.mdpi.com/article/10.3390/cimb44110360/s1. Figure S1: Inverse correlation of miRNA levels and their proposed mRNA targets in GBM according to the data of TCGA project (from GBM-BioDP). Points C, N, M and P denotes expression data of classical, neural, mesenchymal and proneural GBM-subtypes, respectively. Points U—data of unknown GBM subtype according to GBM-BioDP project. The blue dotted line is a linear approximation of the mRNA/miRNA expression data. R2—square of Pearson’s correlation coefficient of the linear approximation. Table S1: Summary table of miRNA and mRNA targets and links to external databases, including GBM-BioDP. Table S2: Links to miRNA expression data in GBM-BioDP. Table S3: Links to mRNA expression data in GBM-BioDP.

## Abbreviations

CNS	central nervous system
CSC	cancer stem cell
GBM	glioblastoma
HC	hierarchical clustering
MN	monolayer
NGS	next generation sequencing
NS	neurosphere
PMID	PubMed identifier
RISC	RNA induced silencing complex
