# Supplementary figures and images for: MicroRNA and mRNA Expression Changes in Glioblastoma Cells Cultivated under Conditions of Neurosphere Formation
Source: Curr Issues Mol Biol. 2022 Oct 30;44(11):5294–311. doi: 10.3390/cimb44110360 (PMC9688839; doi:10.3390/cimb44110360)

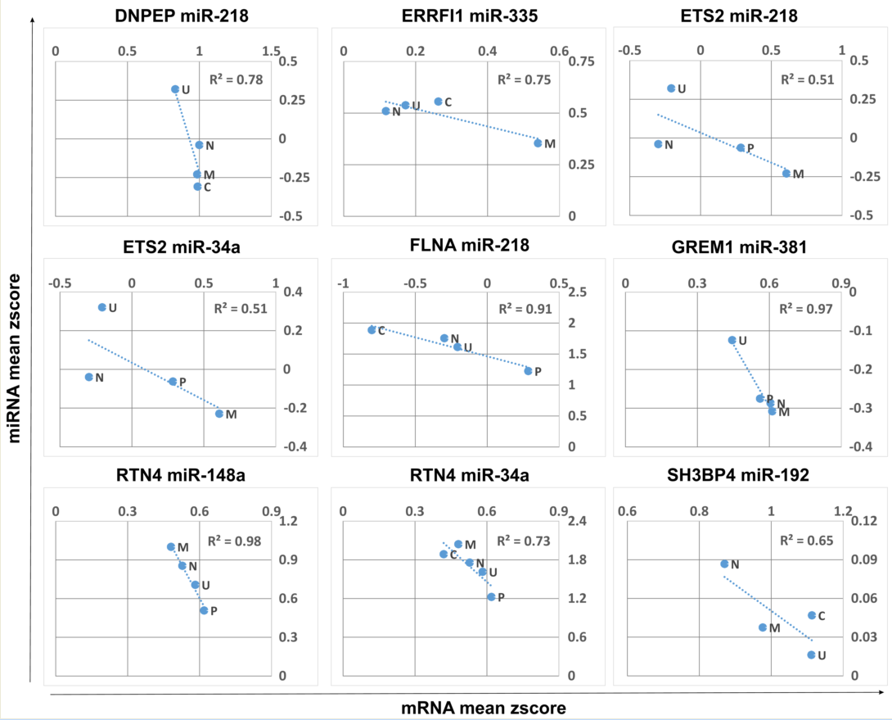

Supplement: Supplementary file 1 [file cimb-44-00360-s001.zip › Figure S1.png]
